# An Early Decrease in Release of Aquaporin-2 in Urinary Extracellular Vesicles After Cisplatin Treatment in Rats

**DOI:** 10.3390/cells8020139

**Published:** 2019-02-10

**Authors:** Hiroko Sonoda, Sayaka Oshikawa-Hori, Masahiro Ikeda

**Affiliations:** Department of Veterinary Pharmacology, Faculty of Agriculture, University of Miyazaki, Miyazaki 889-2192, Japan; sonoda-h@cc.miyazaki-u.ac.jp (H.S.); oshikawa.sayaka.g3@cc.miyazaki-u.ac.jp (S.O.-H.)

**Keywords:** urinary extracellular vesicles, exosomes, aquaporin-1, aquaporin-2, cisplatin, acute kidney injury

## Abstract

Aquaporin-1 (AQP1) and AQP2 are important proteins involved in the regulation of renal water handling. Both AQPs have been found in urinary extracellular vesicles (uEVs) (uEV-AQP1 and -AQP2). Cisplatin, an antineoplastic agent, is known to down-regulate renal AQP1 and AQP2. However, the effect of cisplatin on the release of uEV-AQP1 and -AQP2 is largely unknown. In this study, we examined whether treatment of rats with cisplatin affected the release of uEV-AQP1 and -AQP2. Blood tests indicated that renal function was little altered at 24 h after cisplatin treatment but thereafter decreased dramatically at all of the other time points examined. Release of uEV-AQP1 was slightly increased at 24 h and decreased at 168 h. On the other hand, release of uEV-AQP2 was decreased dramatically at 24 h, and the decrease was maintained during the experimental period. These data suggest that uEV-AQP2 can be used to detect early renal impairment due to cisplatin. Furthermore, a combination of uEV-AQP2 and -AQP1 may be useful for estimation of cisplatin-induced renal injury in a stage-dependent manner.

## 1. Introduction

Aquaporins (AQPs) are membrane water channels, and at least 13 aquaporin isoforms (AQP0 to 12) have been identified in mammals. In the kidney, seven AQPs (AQP1, 2, 3, 4, 6, 7 and 11) are known to be expressed at distinct sites along the nephron [1]. Among them, AQP1 and AQP2 have been identified in the urine. Aquaporin-1 is expressed in the proximal tubule, descending thin limb, and descending vasa recta cells, contributing to water reabsorption in the proximal tubules and formation of a renal osmotic gradient. Aquaporin-2 is expressed mainly at the apical membrane and intracellular vesicles in the collecting duct principal cells. Apical membrane expression of AQP2 is known to be regulated by arginine vasopressin (AVP), an antidiuretic hormone [2].

Urinary extracellular vesicles (uEVs), tiny vesicles produced by renal epithelial cells [3,4], are mainly classified into two subsets including exosomes and microvesicles. Exosomes (30–100 nm in diameter) are released into urine by fusion of the outer membrane of multi-vesicular bodies with the apical plasma membrane of renal epithelial cells [5]. Microvesicles (100–1000 nm in diameter) are produced by a budding process of plasma membrane [5]. Aquaporin-1 and AQP2 in the urine have been reported to be localized to uEVs (uEV-AQP1 and uEV-AQP2), especially exosomes [4,6,7].

We previously reported that uEV-AQP1 and -AQP2 might reflect the expression levels of AQP1 and AQP2 in the proximal tubule and collecting duct cells, respectively [8,9]. Also, the release of uEV-AQP1 and -AQP2 was decreased in models of acute kidney injury (AKI) including gentamicin- and renal ischemia-induced injury models in rats [8,9,10]. 

Cisplatin is a widely used chemotherapeutic agent administered to patients with testicular cancer, ovarian germ cell tumors, and head and neck cancer [11]. However, therapeutic use of cisplatin is associated with toxicity resulting in renal injury [12,13,14]. The proximal tubule in the kidney is known to be the principal site for renal damage due to cisplatin [12,13,14]. It has also been reported that cisplatin treatment causes necrosis of the distal tubules and collecting ducts [12,13,14]. Although the molecular mechanisms underlying cisplatin-induced nephrotoxicity have not been fully elucidated, the toxicity is thought to be mediated through its interaction with nuclear DNA, mitochondrial DNA and other mitochondrial targets. For example, it has been reported that mitochondria in the renal inner medullary collecting duct cells are injured by cisplatin [15,16].

Previous studies using rats have indicated that renal expression of AQP1 and AQP2 is decreased by treatment with cisplatin, resulting in a defect in the urinary concentration mechanism [17,18]. However, it remains unclear whether cisplatin affects the release of uEV-AQP1 and -AQP2. In the present study, we examined the effect of cisplatin on the release of uEV-AQP1 and -AQP2. 

## 2. Materials and Methods

### 2.1. Animal Models

All animal studies were performed with approval from the University of Miyazaki in accordance with Guidelines for the Care and Use of Laboratory Animals in the University of Miyazaki.

Male Sprague-Dawley (SD) rats were purchased from Kyudo (Saga, Japan). All animals were kept in metabolic cages and given free access to water during the study period. Sprague-Dawley rats (316–510 g) were injected with cisplatin (7.5 mg/kg) (Sigma, Tokyo, Japan) intraperitoneally (the cisplatin group). This dose was selected because a previous report had indicated that cisplatin at this dose significantly affected the renal expression of AQPs [18]. Rats receiving only a vehicle (saline) injection served as the control group. Blood and urine samples were collected at 24 h (control, n = 18; cisplatin, n = 18; from 6 experiments), 72 h (control, n = 22; cisplatin, n = 21; from 7 experiments), 120 h (control, n = 15; cisplatin, n = 14; from 5 experiments), and 168 h (control, n = 12; cisplatin, n = 12; from 4 experiments), and kidney samples were obtained at 24 h (control, n = 6; cisplatin, n = 6; from 2 experiments), 72 h (control, n = 10; cisplatin, n = 9; from 3 experiments), 120 h (control, n = 6; cisplatin, n = 5; from 2 experiments), and 168 h (control, n = 6; cisplatin, n = 7; from 2 experiments) after treatment with either vehicle or cisplatin.

Plasma creatinine and urea nitrogen concentrations were measured by an autoanalyzer (FUJIDRICHEM, Fuji Film Medical Co., Ltd. Tokyo, Japan).

### 2.2. Urine Collection and Isolation of uEVs

Urine was collected for 6 h (5 a.m. to 11 a.m.; 24 h, 18–24 h; 72 h, 66–72 h; 120 h, 114–120 h; 168 h, 162–168 h after treatment) with a collection tube containing a protease inhibitor mixture (60 µl of 130 mM EDTA, 70 mM p-amidinophenyl methanesulfonyl fluoride hydrochloride, complete protease inhibitor cocktail tablet). The uEV-rich fraction was obtained using a urine sample whose volume had been adjusted by a certain amount of creatinine (e.g., 1 mg creatinine) employing a sequential differential centrifugation technique (1000× *g* for 15 min at RT, 17,000× *g* for 15 min at 25 °C, 200,000× *g* for 1 h at 25 °C) as described previously [8,9,10]. The pellet obtained after the final centrifugation was suspended in 50 µL of a solution of the above protease inhibitor mixture diluted with MilliQ water. After the suspension was mixed with 4 × sample buffer (8% SDS, 50% glycerol, 250 mM Tris-HCl, 0.05% bromo phenol blue, 200 mM DTT), the mixture was incubated for 30 min at 37 °C.

### 2.3. Kidney Protein Extraction

The kidney was divided into three regions, the cortex, outer medulla and inner medulla, under a stereoscopic microscope. Each region of kidney was homogenized in an ice-cold isolation solution (300 mM sucrose, 1.3 mM EDTA, 25 mM imidazole, complete protease inhibitor cocktail tablet) for 10 min using a shaker-type homogenizer at 1500 rpm for 10 min (Shakemaster Neo, Bio Medical Science, Tokyo, Japan). The homogenate was centrifuged at 1000× *g* for 10 min at 4 °C, and the supernatant was subsequently ultra-centrifuged at 200,000× *g* for 1 h. The pellet obtained from ultra-centrifugation was suspended in the isolation solution, and this suspension was mixed with 4 × sample buffer. This mixture was thereafter incubated at 37 °C for 30 min. The protein concentration in a small amount of suspension solution from each pellet before addition of the sample buffer was determined using the Pierce BCA Protein Assay reagent Kit (Thermo Fisher Scientific Inc., Rockford, IL, USA).

### 2.4. Immunoblot Analysis

Immunoblot analysis was performed as previously described [8,9,10], using the following antibodies: Anti-AQP1 (cat no. sc-20810; Santa Cruz Biotechnology Inc., Santa Cruz, CA, USA), anti-AQP2 (cat no. AQP-002; Alomone Labs, Jerusalem, Israel), anti-GAPDH antibody (cat. no. sc-25778; Santa Cruz Biotechnology Inc.), and HRP-conjugated anti-rabbit IgG (cat no. 7074; Cell Signaling Technology, Danvers, MA, USA). Antibody-associated protein on the membrane was detected by Super Signal^®^ chemiluminescence detection system (Thermo Fisher Scientific Inc.). The protein bands were visualized by a polaroid camera (GE Healthcare UK Ltd., Amersham, England) or a LAS4000 system (GE Healthcare UK Ltd.). The pictures taken by the camera were scanned using a scanner (GT-S650, Seiko Epson corp., Nagano, Japan) and the density of the band was quantified by the WinRoof software V5.7 (MITANI CORPORATION, Tokyo, Japan). The representative picture taken by the camera was shown after a monochrome inversion under the Adobe Photoshop CC 2017 software (ver 18.0.1, Adobe Systems Co., Ltd, Tokyo, Japan), while retaining the original quality. The resulting band visualized by the LAS4000 system was evaluated by a ImageQuant TL software (GE Healthcare UK Ltd.).

For preliminary validation of the GAPDH internal control, the levels of renal expression of GAPDH were compared between the control and cisplatin groups. The mean ± standard error of the mean (SEM) values are shown in a supplementary table (Table S1), and the differences in values between the groups for the same region at each time point were not significantly different, indicating that GAPDH was appropriate as an internal control.

In each series of experiments, a control group comprising several animals was included. When immunoblotting analysis was performed, protein samples from the corresponding control animals were always loaded in each gel for normalization.

### 2.5. Histology

The paraffin-embedded kidney blocks (fixation with 10% paraformaldehyde) were cut at 2 µm thickness and the sections were stained with periodic acid-Schiff (PAS) reagent (Muto Pure Chemicals Co., Ltd., Tokyo, Japan).

For immunofluorescence staining, after retrieval of antigen by incubating specimen in distilled water at 121 °C for 5 min, the specimens were immersed in a 3% H_2_O_2_ solution to consume the endogenous peroxidase and then were blocked with 1% bovine serum albumin for 15 min. After washing, the specimens were incubated with ani-AQP2 antibody for 45 min at 37 °C. Then, the specimens were exposed to secondary antibody, Alexa Fluor 488-conjugated chicken anti-rabbit IgG (cat. no. A31571; Invitrogen, Life Technologies, Carlsbad, CA, USA). Microscopic observation was performed using a confocal microscope (Fluoview FV300, Olympus, Tokyo, Japan). 

### 2.6. Statistical Analysis

The data are shown as a box plot. Individual values are represented as dots. Also, the mean ± SEM values are shown in supplementary tables (Tables S2 and S4–S6). 

Statistical comparisons were accomplished by Mann–Whitney U test. *p* values < 0.05 were considered statistically significant.

## 3. Results

### 3.1. Kidney Injury After Treatment with Cisplatin 

**Figure 1:**
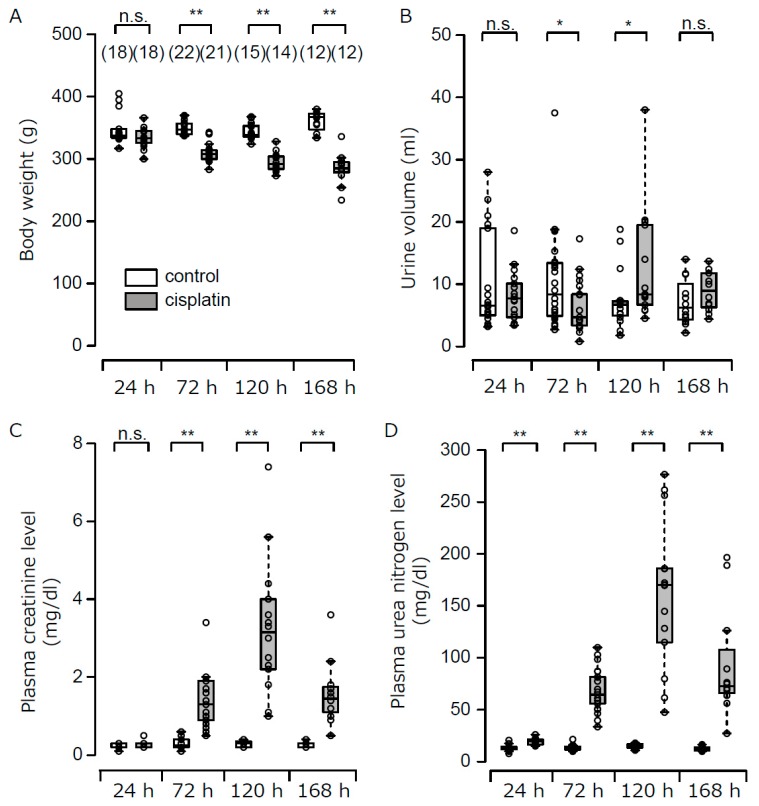
Time course of changes in body weight, urine volume, plasma creatinine and urea nitrogen concentrations after cisplatin treatment. Body weight (**A**), urine volume (**B**), plasma creatinine (**C**), and urea nitrogen concentrations (**D**) at 24 h, 72 h, 120 h, and 168 h after treatment with either vehicle or cisplatin are shown. Numbers in parentheses indicate the number of animals subjected to experiments. * *p* < 0.05, ** *p* < 0.01, vs control rats. n.s. represents no significance.

The summarized data for body weight, urine volume, plasma creatinine, and urea nitrogen concentrations are shown in Figure 1 and Table S2. Although body weight in the control group was not altered during the experimental period, that in the cisplatin group gradually decreased. Urine volume in the cisplatin group significantly decreased at 72 h and increased at 120 h in comparison with the control group. Cisplatin caused significant increases in plasma creatinine and urea nitrogen concentrations at 72 h, 120 h, and 168 h, and the highest values were observed at 120 h. On the other hand, at 24 h, cisplatin had no significant effect on the plasma creatinine concentration, although it caused a small but significant increase in the plasma urea nitrogen concentration. However, both values were within the normal range for rats, based on data provided by the laboratory animal production and supply company (Japan SLC Inc., http://jslc.co.jp/pdf/rat/001_SD2013.pdf) (Figure 1C,D). 

**Figure 2:**
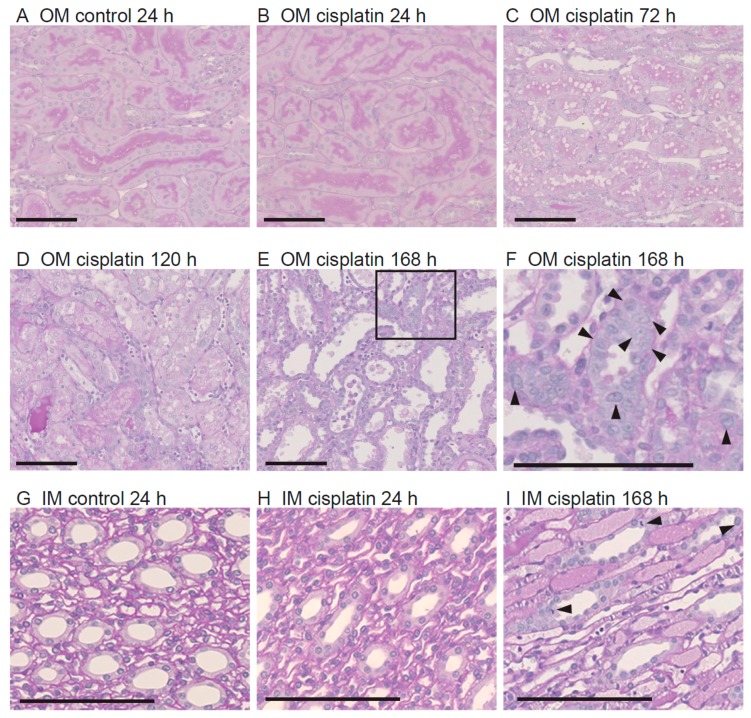
Renal histology after treatment with cisplatin. The outer (**A**–**F**) and inner medulla (**G**–**I**) regions of kidney sections stained with periodic acid-Schiff (PAS) from a rat in the control group at 24 h (**A**) and (**G**) and from rats in the cisplatin group at 24 h (**B**) and (**H**), 72 h (**C**), 120 h (**D**), and 168 h (**E**), (**F**) and (**I**) are shown. These pictures are representative of three individuals at each time point and treatment. The high-magnification image of the box in (**E**) is shown in (**F**). Arrow heads indicate the regenerating or mitotic tubular cells. Bars = 100 µm.

As shown in Figure 2B, no histological change was observed in the outer medulla of the cisplatin group at 24 h after the treatment. However, vacuolization of proximal tubular cells and disruption of brush border were observed at 72 h (Figure 2C). At 120 h, cisplatin caused loss of brush border, tubular cell necrosis, occlusion of tubular lumen by necrotic cell debris and casts, and cell infiltration in the interstitial region (Figure 2D). At 168 h, in addition to a loss of brush border, cellular infiltration, and necrotic cell debris, remarkable dilation of tubular lumen and tubular cell regeneration (arrowheads in Figure 2F) were also observed in the outer medulla (Figure 2E,F). In the inner medulla, treatment with cisplatin for 24 h had little effect on the histology (Figure 2H). However, at 168 h after the treatment, tubule dilation and cast formation were evident, especially in the loop of Henle (Figure 2I). Also, cells showing regeneration and mitosis (arrowheads in Figure 2I) were observed in the inner medulla.

### 3.2. Release of uEV-AQP1 and -AQP2 after Treatment with Cisplatin 

Next, we examined the release of uEV-AQP1 after cisplatin treatment. We first measured urinary creatinine excretion in the control and cisplatin groups, and the mean ± SEM values were shown in Table S3. In comparison with the control group, cisplatin had no significant effect on the excretion of creatinine at 24 h, 120h, and 168 h. At 72 h, cisplatin caused a slight decrease in the excretion by approximately 70%. These data suggested that when the level of urinary creatinine was used to normalize the level of protein in the uEV, this normalization method was appropriate at the 24 h, 120 h, and 168 h time points. Also at 72 h, even if a normalization error was present, in most cases, it would have been less than 30%. Furthermore, this contamination error would have led to high estimates of the protein levels in the uEV. When the data show a significant decrease in the level of protein in the uEV, it is considered that the real value would be lower. 

**Figure 3:**
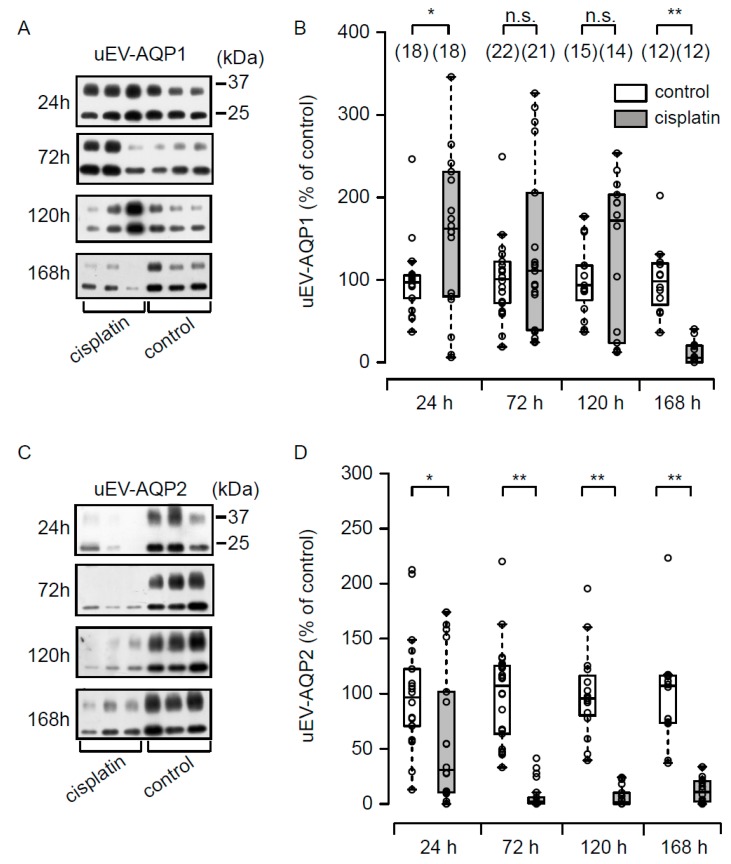
Release of aquaporin-1 in urinary extracellular vesicles (uEV-AQP1) and -AQP2 after cisplatin treatment. Typical immunoblots for uEV-AQP1 (**A**) and uEV-AQP2 (**C**) are shown. The summarized quantified data of immunoblot analyses of uEV-AQP1 (**B**) and uEV-AQP2 (**D**) are shown as boxplot graphs. Each value is indicated as a percentage of the mean value of uEV-AQP1 or -AQP2 level in the control rats at each time point. * *p* < 0.05, ** *p* < 0.01, vs control rats. n.s. represents no significance. The loading volume for immunoblot was normalized to urinary creatinine content.

As shown in Figure 3A,B (Table S4), release of uEV-AQP1 in the cisplatin group was significantly increased at 24 h. At 72 h and 120 h, the difference between the control and cisplatin groups was not significant. At 168 h, release of uEV-AQP1 was significantly decreased. 

Figure 3C,D (Table S4) summarizes the data for uEV-AQP2. Release of uEV-AQP2 in the cisplatin group was significantly decreased even at 24 h, and the decrease was maintained during the experimental period.

### 3.3. Renal Expression of AQP1 and AQP2 after Treatment with Cisplatin 

**Figure 4:**
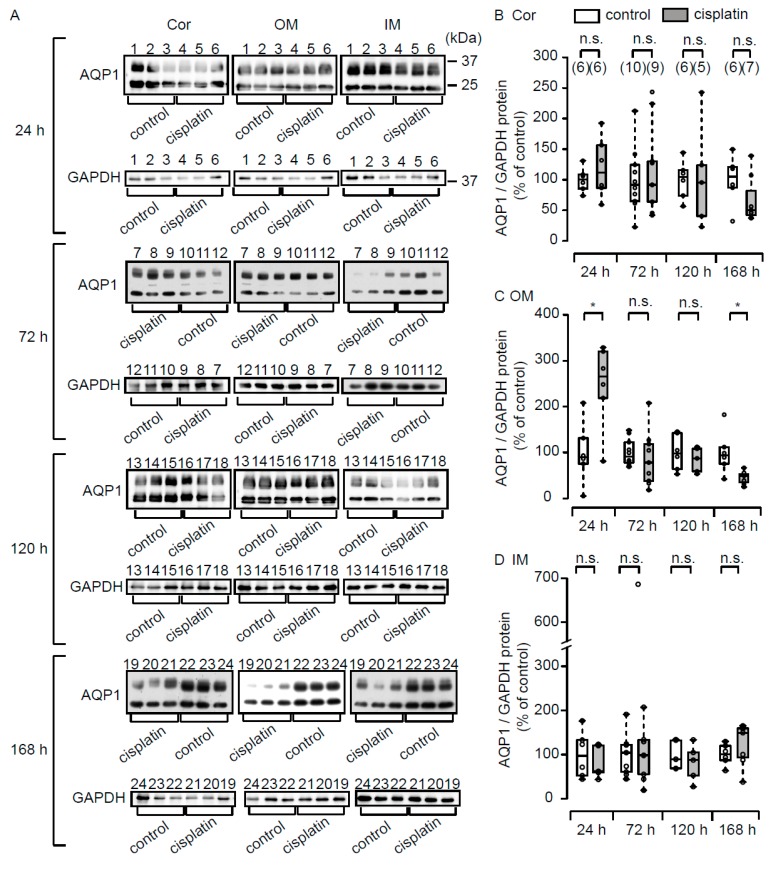
Renal AQP1 expression after cisplatin treatment. Representative immunoblots of renal AQP1 and GAPDH (**A**) and the quantified data for the cortex (Cor) (**B**), the outer medulla (OM) (**C**), and the inner medulla (IM) (**D**) are shown. Aquaporin-1 protein levels were normalized with corresponding GAPDH levels. Each value is expressed as percentage of mean value of the AQP1 levels in the control group at the corresponding time points. * *p* < 0.05, vs control rats. n.s. represents no significance. The number above each lane indicates the individual rat number. Each lane was loaded with the same amount of total protein in the same gel.

Renal expression level of AQP1 was evaluated by immunoblotting, and the results are shown in Figure 4 (Table S5). Expression levels of AQP1 in the cortex and inner medulla in the cisplatin group were not significantly different from those in the control group at all of the time points examined in this study. On the other hand, in the outer medulla, cisplatin significantly increased expression of AQP1 at 24 h and decreased it at 168 h. 

**Figure 5:**
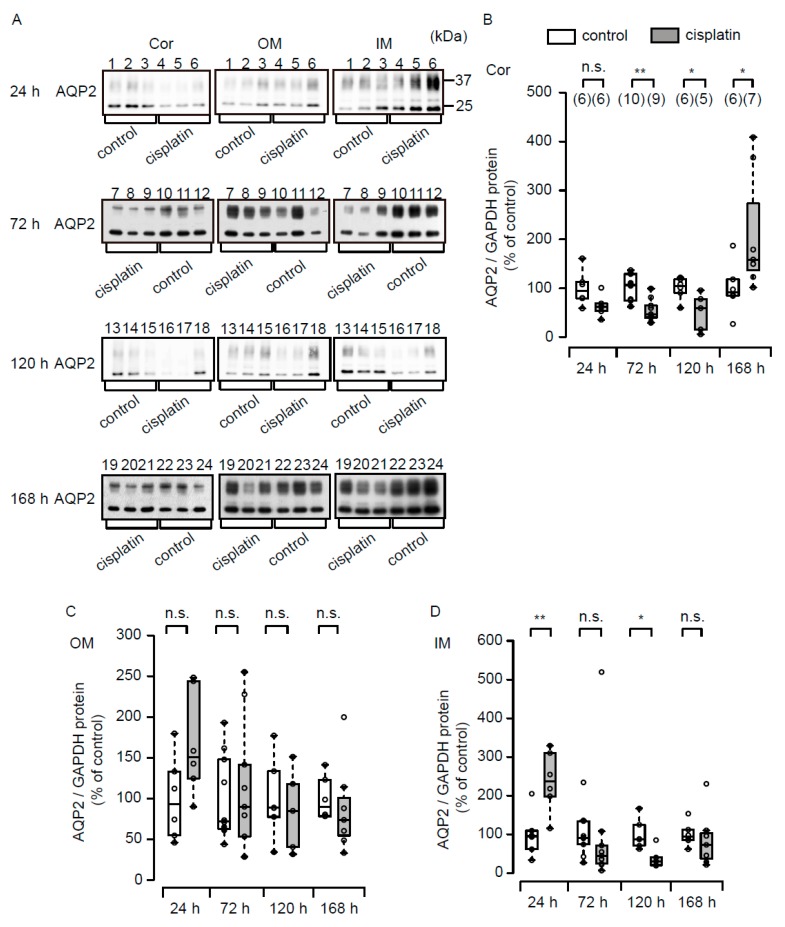
Renal AQP2 expression after cisplatin treatment. Representative immunoblots of renal AQP2 (**A**) and the quantified data for the cortex (Cor) (**B**), the outer medulla (OM) (**C**), and the inner medulla (IM) (**D**) are shown. Aquaporin-2 protein levels were normalized with corresponding GAPDH levels (shown in Figure 4). Each value is expressed as percentage of mean value of the AQP2 levels in the control group at the corresponding time points. * *p* < 0.05, ** *p* < 0.01, vs control rats. The number above each lane indicates the individual rat number. n.s. represents no significance. Each lane was loaded with the same amount of total protein in the same gel.

Figure 5 (Table S6) summarizes the data for renal expression of AQP2. In the cortex, expression of AQP2 was significantly decreased at 72 h and 120 h in the cisplatin group in comparison with the control group. In contrast to these decreases, at 168 h cisplatin up-regulated the expression of AQP2. In the outer medulla, cisplatin had little effect on the expression of AQP2 during the experimental period. In the inner medulla, expression of AQP2 was significantly increased at 24 h. On the other hand, the expression was decreased at 72 h, 120 h and 168 h, and the difference at 120 h was significant.

**Figure 6:**
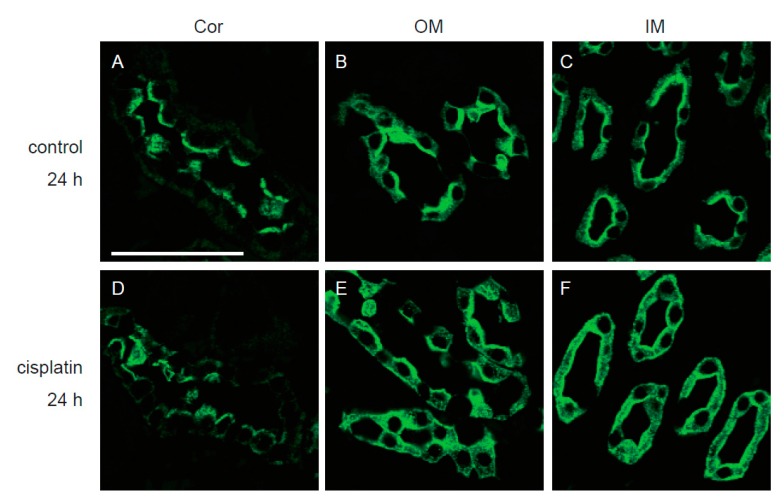
Immunofluorescence examination of renal AQP2 at 24 h after cisplatin treatment. The cortex (**A**) and (**D**), outer medulla (**B**) and (**E**), and inner medulla (**C**) and (**F**) regions of kidney sections stained with anti-AQP2 antibody at 24 h after treatment with either vehicle (**A**–**C**) or cisplatin (**D**–**F**). Bar = 50 µm.

As mentioned earlier, release of uEV-AQP2 was markedly decreased at 24 h after cisplatin treatment, even though the histological change was minimal. Therefore, we performed an immunofluorescence study using a sample from a rat that exhibited lower release of uEV-AQP2 (10.3% relative to the control). As shown in Figure 6, there were no marked differences in the expression of AQP2 in the cortex and outer medulla between the control and cisplatin groups. In the inner medulla, expression of AQP2 was somewhat increased at 24 h after treatment with cisplatin, especially in the intracellular region.

## 4. Discussion

In the present study, the plasma creatinine and urea nitrogen concentrations were within the normal range at 24 h after cisplatin treatment. Thereafter, these concentrations increased progressively until 120 h, and then were decreased somewhat at 168 h. In accordance with these alterations, histological analyses showed that renal injury was minimal at 24 h, whereas at 120 h loss of brush border, tubule cell necrosis, occlusion of the tubule lumen, and cell infiltration in the interstitial region were observed. At 168 h these pathological changes were still evident, and also cells characterized by a large nucleus, having an irregular size and shape, and mitotic figures were observed, suggesting that some of them were in the growth phase. This time course of cisplatin-induced renal injury corroborated previous reports [19]. During this experimental period, release of uEV-AQP1 was significantly increased at 24 h, and thereafter returned to the control level. At 168 h, release of uEV-AQP1 was markedly decreased. On the other hand, release of uEV-AQP2 was significantly decreased even at 24 h and this lower level was maintained until 168 h. These results suggest that release of uEV-AQP2 might be a better indicator of early to late renal injury after cisplatin treatment. Furthermore, uEV-AQP1 and uEV-AQP2 in combination may reflect the later phase of cisplatin-induced kidney injury more accurately.

An interesting observation in this study was the early and continuous decrease in the release of uEV-AQP2 after cisplatin treatment. It has been suggested that the level of uEV-AQP2 is regulated by the abundance of its renal protein. For example, in rats with gentamicin-induced nephrotoxicity [8], release of uEV-AQP2 was significantly reduced, accompanied by a significant reduction of its renal expression. In the present study, expression of AQP2 was decreased in the cortex at 72 h and 120 h, and in the inner medulla at 120 h, after cisplatin treatment. Therefore, it is considered that the decreased renal expression in these regions is involved in the reduction of uEV-AQP2 release at 72 h and 120 h after cisplatin treatment. However, since cisplatin did not significantly reduce the expression of AQP2 protein at 24 h, the decreased level of renal expression would not have explained the altered release of uEV-AQP2 at 24 h. Besides the renal abundance of AQP2, it has been suggested that AVP may be another factor regulating the release of uEV-AQP2. Wen et al. [3] have shown that treatment of rats with desmopressin, an AVP analogue, caused a three- to four-fold increase in excretion of uEV-AQP2 in an apical-expression-dependent manner. Dear’s group has reported that treatment of mCCDc11 cells, a kidney collecting duct cell line, with desmopressin increases the secretion of EV-AQP2 into the culture medium [20]. In rats, Clifton et al. have observed a decrease in the secretion of AVP from the posterior pituitary within 24 h after the cisplatin treatment [21]. Therefore, the inhibitory effect of cisplatin on the release of uEV-AQP2 at 24 h may be due to reduced apical membrane expression of AQP2 via a decrease in the secretion of AVP. The increased intracellular expression of AQP2 we observed in the inner medulla (Figure 6F) may support this notion. Although it has been reported that direct measurement of the plasma AVP concentration is difficult, due to the association of AVP with platelets and the short half-life of plasma AVP, the measurement of a surrogate AVP marker, such as copeptin, might help to clarify the mechanism [22,23,24].

In the present study, we observed that release of uEV-AQP1 was significantly increased at 24 h and decreased at 168 h after cisplatin treatment. Along with these observations, cisplatin significantly increased the expression of AQP1 in the outer medulla at 24 h and decreased it at 168 h. Because, similarly to AQP2, renal expression of AQP1 has also been reported to contribute to the release of uEV-AQP1 [9], alteration in the release of uEV-AQP1 is thought to be mediated through changes in its level of expression in the outer medulla. 

We showed that expression of AQP1 in the outer medulla was significantly decreased and that of AQP2 in the inner medulla tended to be decreased at 168 h after cisplatin treatment. Similar observations have been made in the later phase of many types of renal injury models, including ischemia/reperfusion and gentamicin-treatment models [8,9,10]. In this phase, cell proliferation is thought to repair the injury sites. In fact, in the present study, we observed some cells in the growth phase at this time point. Also, markers of cell proliferation, including proliferating cell nuclear antigen, are reportedly expressed in a similar phase in other experimental models of renal injury [25,26]. On the other hand, in growing renal cells, it has been considered that expression of renal functional proteins such as transporters and AQPs are down-regulated [27,28]. Therefore, the increase in the number of proliferative cells may result in reduction of the renal expression of AQP1 and AQP2 at a later phase of cisplatin-induced kidney injury.

## 5. Conclusions

We have found that cisplatin affects the release of uEV-AQP1 and -AQP2, and that examination of these uEV-AQPs in combination may allow early to late detection of cisplatin-induced renal impairment. However, as studies on the regulation of uEV-AQP1 and -AQP2 release are still in their early stages, further work to identify the molecular mechanism underlying their release will be necessary.

## Supplementary Materials

The supplementary materials are available online https://www.mdpi.com/2073-4409/8/2/139/s1.
