# Supplementary material for: An Early Decrease in Release of Aquaporin-2 in Urinary Extracellular Vesicles After Cisplatin Treatment in Rats
Source: Cells. 2019 Feb 10;8(2):139. doi: 10.3390/cells8020139 (PMC6407024; doi:10.3390/cells8020139)
Supplement: Supplementary file 1 [file cells-08-00139-s001.pdf]

Table S1. The mean ± standard error of the mean (SEM) values for GAPDH.

Renal GAPDH		24 h	72 h	120 h	168 h
N	control	6	10	6	6
	cisplatin	6	9	5	7
Cor	control	100.0 ± 12.1	100.0 ± 9.9	100.0 ± 7.9	100.0 ± 20.0
	cisplatin	75.1 ± 13.7	111.6 ± 12.8	132.3± 23.3	74.3 ± 8.1
OM	control	100.0 ± 9.1	100.0 ± 9.1	100.0 ± 15.6	100.0 ± 8.5
	cisplatin	77.0 ± 14.8	92.2 ± 12.1	108.7 ± 25.8	106.2 ± 14.8
IM	control	100.0 ± 15.3	100.0 ± 17.2	100.0 ± 7.1	100.0 ± 13.7
	cisplatin	78.2 ± 10.1	90.8 ± 23.8	109.7 ± 10.1	89.6 ± 6.1

Table S2. The mean ±SEM values for Figure 1.

		24 h	72 h	120 h	168 h
N	control	18	22	15	12
	cisplatin	18	21	14	12
Body weight (g)	control	346.2 ± 5.6	349.5 ± 2.2	343.7 ± 3.3	360.8 ± 4.5
	cisplatin	334.4 ± 3.6	310.6 ± 3.4**	295.1 ± 4.0**	284.8 ± 7.2**
Urine volume (ml)	control	10.3 ± 1.9	10.4 ± 1.7	7.5 ± 1.3	7.1 ± 1.1
	cisplatin	8.0 ± 1.0	6.4 ± 0.9*	12.6 ± 2.4*	8.9 ± 0.9
Plasma creatinine (mg/dl)	control	0.2 ± 0.01	0.3 ± 0.03	0.3 ± 0.02	0.3 ± 0.02
	cisplatin	0.3 ± 0.02	1.4 ± 0.1**	3.3 ± 0.5**	1.6 ± 0.2**
Plasma urea nitorogen (mg/dl)	control	13.6 ± 0.7	13.2 ± 0.6	15.0 ± 0.6	12.4 ± 0.6
	cisplatin	19.5 ± 0.7**	68.5 ± 4.5**	161.0 ± 19.2**	92.3 ± 15.0**
		*; P < 0.05, **; P < 0.01 vs control			

Table S3. The mean ±SEM values for urinary excretion of creatinine.

Urinary total creatinine					
		24 h	72 h	120 h	168 h
N	control	18	22	15	12
	cisplatin	18	21	14	12
Urinary total creatinine (mg/6h)	control	4.7 ± 0.5	5.1 ± 0.5	3.7 ± 0.4	3.1 ± 0.3
	cisplatin	4.2 ± 0.3	3.5 ± 0.5*	4.7± 0.6	2.8 ± 0.3
		*; P < 0.05 vs control			

Table S4. The mean ±SEM values for Figure 3.

		24 h	72 h	120 h	168 h
N	control	18	22	15	12
	cisplatin	18	21	14	12
uEV-AQP1	control	100.0 ± 10.7	100.0 ± 10.2	100.0 ± 11.1	100.0 ± 12.4
	cisplatin	158.1 ± 22.5*	156.0 ± 34.8	131.5 ± 24.8	12.2 ± 4.1**
uEV-AQP2	control	100.0 ± 12.5	100 ± 9.7	100 ± 10.5	100.0 ± 14.0
	cisplatin	61.2 ± 14.7*	7.4 ± 2.8**	6.4 ± 2.4**	12.2 ± 3.2**
		*; P < 0.05, **; P < 0.01 vs control			

Table S5. The mean ±SEM values for Figure 4.

Renal AQP1		24 h	72 h	120 h	168 h
N	control	6	10	6	6
	cisplatin	6	9	5	7
Cor	control	100.0 ± 8.1	100.0 ± 16.5	100.0 ± 12.7	100.0 ± 16.4
	cisplatin	119.6 ± 20.3	117.8 ± 24.3	105.2 ± 39.0	67.8 ± 14.9
OM	control	100.0 ± 27.4	100.0 ± 9.2	100.0 ± 15.8	100.0 ± 19.1
	cisplatin	246.7 ± 37.3*	86.7 ± 19.9	83.9 ± 12.1	45.2 ± 5.4 *
IM	control	100.0 ± 21.4	100.0 ± 13.9	100.0 ± 12.1	100.0 ± 9.8
	cisplatin	78.1± 14.0	163.4± 68.0	84.1 ± 18.8	122.6 ± 18.4
		*; P < 0.05 vs control			

Table S6. The mean ±SEM values for Figure 5.

Renal AQP2		24 h	72 h	120 h	168 h
N	control	6	10	6	6
	cisplatin	6	9	5	7
Cor	control	100.0 ± 14.7	100.0 ± 9.0	100.0 ± 9.3	100.0 ± 21.4
	cisplatin	63.8 ± 8.8	54.9 ± 7.9 **	50.9 ± 17.5*	212.6 ± 46.5*
OM	control	100.0 ± 21.0	100.0 ± 16.3	100.0 ± 20.2	100.0 ± 10.9
	cisplatin	168.1 ± 26.4	113.1 ± 27.3	85.3 ± 22.7	88.3 ± 21.1
IM	control	100.0 ± 23.9	100.0 ± 18.4	100.0 ± 16.0	100.0 ± 12.7
	cisplatin	237.7 ± 32.0**	98.7 ± 53.5	39.2 ± 12.1*	86.6 ± 27.1
		*; P < 0.05, **; P < 0.01 vs control			
